# Late Systemic Lupus Erythematosus-Associated Insulin Resistance Syndrome: A Rare Cause of De Novo Diabetes Mellitus

**DOI:** 10.1155/2022/4655804

**Published:** 2022-10-14

**Authors:** José C. Alvarez-Payares, Daniel Ribero, Luis Rodríguez, Carlos E. Builes, Carolina Prieto, Clara Arango, Juan G. Gamboa, Cristian Alvarez-Payares

**Affiliations:** ^1^Department of Internal Medicine, Universidad de Antioquia, Medellin, Colombia; ^2^Department of Endocrinology and Metabolism, Hospital Pablo Tobon Uribe, Medellín, Colombia; ^3^Department of Internal Medicine, Hospital Pablo Tobon Uribe, Medellín, Colombia; ^4^Universidad de Sucre, Sincelejo, Colombia

## Abstract

The association of type B insulin resistance syndrome (TBIRS) due to autoimmune diseases such as systemic lupus erythematosus (SLE) is uncommon. This is partly due to the lack of established criteria for the diagnosis of this resistance. However, some clinical aspects may suggest that the diagnosis does not necessarily have to be positive insulin receptor antibodies as such patients could respond to immunosuppressive treatment. *Methods*. We describe a case and have performed a literature review on PubMed/MEDLINE, EMBASE, and Google Scholar bibliographic databases to identify all case reports. All available studies from January 1975 through December 2020 were included. Data collected were tabulated, and outcomes were analyzed cumulatively. *Results*. Thirty-one cases of TBIRS associated with SLE have been described. These patients presented with catabolic symptoms and hyperglycemia in most cases, with an average time from the onset of symptoms of four months. In addition to that clinical characteristics related to SLE were variable, along with certain common characteristics such as acanthosis in 60% of patients. Almost all the patients had antibodies against insulin receptors. The insulin doses required by the patients ranged from 450 to 25,000 U daily. Remission was achieved in 80% of the patients with a two-year follow-up. Most patients associated with late-onset SLE, like our patient, achieved metabolic control after immunosuppressive treatment. *Conclusion*. High insulin resistance in patients with de novo diabetes mellitus (DM) without obesity should be considered as a possible clinical manifestation of an autoimmune disease such as SLE, with a good metabolic response to the immunosuppressive management established.

## 1. Introduction

Type B insulin resistance syndrome (TBIRS) is a rare autoimmune disease mediated by autoantibodies directed against insulin receptors, leading to hyperglycemia secondary to severe insulin resistance; however, it can lead to hypoglycemia as well [1]. The cases recorded in the literature typically show women of reproductive age, mainly with systemic lupus erythematosus (SLE) in up to 33% [2]. Patients usually present with weight loss, hyperandrogenism, diffuse acanthosis nigricans associated with insulin resistance, hyperadiponectemia, and hypotriglyceridemia. No standardized treatment exists for this syndrome; however, multiple treatment schemes with variable success rates have been described, including spontaneous remission in up to 33% of patients [3]. Mortality rates can be as high as 50% and the cause of death is usually related to hypoglycemia complications [2].

In the present case report, an elderly nonobese patient with de novo diabetes mellitus diagnosis, high insulin requirement since diagnosis, and inadequate metabolic control is described. The systematic workup of differential diagnosis led to the finding of an autoimmune disease which explains the clinical picture of the patient. In addition, an exhaustive review of SLE-associated TBIRS cases is performed.

## 2. Methods

**Figure 1:**
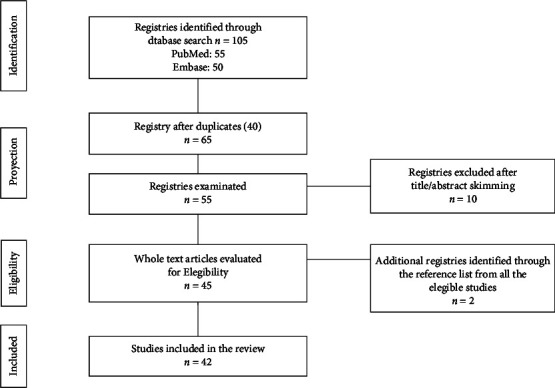
Flowchart describing the cases selection process.

A search was performed in PubMed/Medline, EMBASE, and Google Scholar from January 1975 until March 2021, using the following keywords: “Type B insulin resistance” *o* “Type B syndrome,” “SLE,” “Lupus,” and “Lupus Erythematosus Systemic” *y* “autoimmune.” From 55 articles found (Figure 1), we included every single one when the complete text was available, regardless of the methodology used. Therefore, patients with a confirmed disease or even those with no anti-insulin antibodies but a suggestive clinical presentation were included. We included articles in English, Spanish, or Portuguese. The clinical, demographical, laboratory, treatment, and relapse data were analyzed.

## 3. Case Presentation

**Figure 2:**
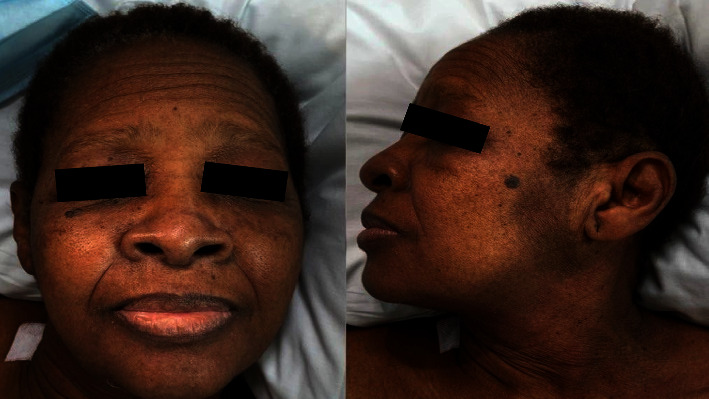
Periocular, perioral, cervical, and mandibular acanthosis.

A 62-year-old African American female, with a personal history of de novo diabetes mellitus with insulin requirements, of two to three months, with no micro or macrovascular complications, treated with high insulin doses (50 U glargine twice a day, and 20 U glulisine three times a day), without achievement of metabolic control. The patient was referred to a fourth-level hospital based on a malignant neoplasm suspicion: weight loss of 20–30 kg in the last two to three months (her weight at the time of evaluation was 100 kg) and absence of metabolic control. The patient mentioned occasional Raynaud's phenomenon. Periocular and perioral acanthosis nigricans (Figure 2), and a left supraclavicular mobile lymphadenopathy of 1 cm diameter, with no inflammatory changes, were observed in physical examination. The rest of the physical examination was unremarkable. During in-patient stance, glucometer registry was between 350–400 mg/dL, despite receiving glargine insulin 138 U/day and glulisine insulin 44 U three times a day.

In Table 1, the main laboratory findings are registered, including lymphopenia and positive antinuclear antibodies (ANA) that associated with Raynaud's phenomenon, suggested a late-onset systemic lupus erythematosus (SLE) diagnosis. However, as a malignant neoplasm was suspected, a cervical lymph node biopsy and a bone marrow biopsy were performed, which were unremarkable. The rest of the endocrine and imaging tests were normal.

**Figure 3:**
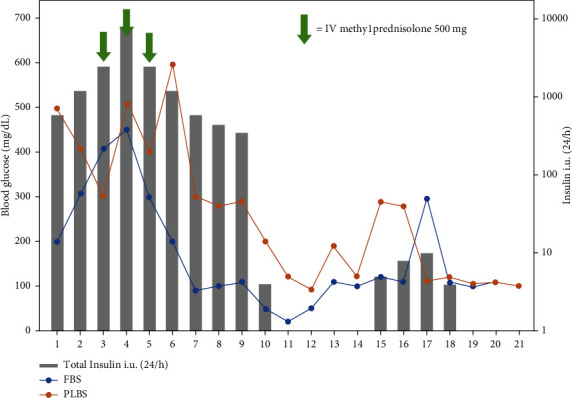
Blood glucose plot. Improvement after IV methylprednisolone pulse and oral prednisolone therapy.

Due to the high insulin requirements, an insulin drip was started and titrated, with the highest dose up to 43 U/kg/day with capillary blood glucometer around 200 mg/dL. An insulin resistance syndrome mediated by autoimmunity was suspected based on the clinical presentation–in this case, late-onset SLE. Therefore, management with methylprednisolone 250 mg/day for three days and a prednisolone taper starting at 50 mg/day was initiated. Insulin dose was successfully reduced to less than 2 U/kg/day and a bridge with subcutaneous insulin was performed until insulin drip was suspended (Figure 3). Nonetheless, the patient presented catheter-associated bacteremia, which delayed rituximab initiation. Rituximab therapy was finally started, achieving excellent clinical and metabolic results because at discharge, she only required linagliptin for glycemic control without the need for insulin or another hypoglycemic agent. The patient remained on maintenance immunosuppression with prednisolone 5 mg and chloroquine 250 mg daily. Unfortunately, after discharge, she did not return to our institution for reasons related to her health insurance.

## 4. Discussion

Type B insulin resistance syndrome (TBIRS) is an extremely rare entity, with unknown prevalence. Only 116 cases have been described in the literature [2, 4]. The first time this disease was described was in 1975 when six patients presented with overt insulin resistance with acanthosis nigricans and high insulin requirements due to a serum-circulating factor which affected insulin binding to its receptor [5]. These patients required up to 100 times more insulin. Extreme requirements from 700 up to 177,500 insulin units per day were registered [6].

Some authors like Willard et al. [1], suggest that the biochemical triad of extremely high levels of fasting insulin, hyperadiponectinemia, and hypotriglyceridemia in patients with acanthosis nigricans and underlying autoimmune disease may be considered as a clinical definition of TBIRS. In addition, other characteristics may lead to TBIRS suspicions, such as slim patients with insulin requirements higher than 3 U/kg/day and persistent hyperglycemia [7].

Three mechanisms have been described for hyperglycemia in this disorder [1]: autoantibodies competing for the insulin receptor binding site, the binding of such autoantibodies leading to receptor degradation, and the agonist/antagonist action of these autoantibodies with a biphasic response (hypo and hyperglycemia).

The biggest TBIRS cohort was obtained in the National Institutes of Health (NIH). In this cohort, 24 patients were followed for 28 years, finding that TBIRS is more frequent in African American women, with an underlying autoimmune disease, and an age between 20 and 68 years, although teenager cases have been described [3]. In these patients, only three of them developed hypoglycemia, and SLE was observed in up to 46% of patients. Most had a BMI less than 30 kg/m^2^ and in those with a BMI lower than 25 kg/m^2^, one third presented with hypoglycemia and two thirds with hyperglycemia, reflecting an insulin resistance profile different to those with obesity. Perioral and periocular acanthosis nigricans were observed in up to 88% of patients; other patients presented with a deeper voice and lower extremity wasting due to overweight. In the autoimmune laboratory, ANA was observed in 83% of patients and hypocomplementemia (C3 predominantly) in 21%. 25% had spontaneous remission and patients treated with glucocorticoids, cyclophosphamide, plasmapheresis, cyclosporine, and azathioprine had a variable response time from five months up to 54 months.

In a recent systematic review [2], 115 TBIRS cases were reported. Most were women (76.5%) with a mean age of 42 years. 50% had normal weight and acanthosis nigricans, 45% had hyperglycemia, 42.9% had hypoglycemia at any time of the disease course, and diabetic ketoacidosis was observed in only 11.8%. SLE was the main etiology in 33%, as the NIH cohort. In some patients with Hodgkin lymphoma and multiple myeloma, TBIRS was the initial manifestation as a paraneoplastic phenomenon. In relation to laboratory findings, mean HbA1c was 10.8% (5.1–18.7), fasting serum insulin of 1309 *μ*g/dl (0.1–10.584), C peptide of 13.9 ng/mL (0.1–63.0), and triglycerides of 72.8 mg/dL (36–155); autoimmunity laboratory was remarkable for ANA, present in 60%, hypocomplementemia in 20%, and anti-insulin antibodies were ordered in only 36.1% with half being positive.

Out of 115 patients, 83 (70%) achieved disease remission and 20.5% with spontaneous remission. In the first phase, prednisolone was used in 40% of patients (dose of 50–60 mg), cyclophosphamide in 20%, rituximab in 10%, and plasmapheresis in 8–10%. During this phase, remission was achieved in 40% of patients. The mean daily insulin dose was 1747 U/day (54–57.600), which led to admission for intravenous drip titration. Time-to-remission had a mean of four months (0.25–54). No statistically significant relationship could be established between negative anti-insulin antibodies and disease remission. The mortality in this systematic review was 15.38%; one out of four patients died due to intractable hypoglycemia.

Thirty-one SLE-associated TBIRS cases have been described (Tables 2 and 3), these patients had hypoglycemia (35%) or catabolic symptoms and hyperglycemia (66%) as the initial symptoms. The time of symptom onset was from one up to 15 months. The most common ANA pattern was speckled (32%). No single SLE sign or symptom was predominant, as the clinical presentation was variable among all patients. Acanthosis nigricans was present in 60% of patients. Predominant antibodies were anti-insulin receptors (97%). 37.5% TBIRS were associated with late-onset SLE. Insulin doses required were between 450–25,000 U/day. Remission was achieved in 80% of patients with a follow-up of two years. 10% of patients did not respond to the therapy, with persistent hypoglycemia. Most late-onset SLE-associated TBIRS required additional immunosuppressant therapy to achieve remission and metabolic control (other than steroids). Nonetheless, in our patient glycemic control was achieved after glucocorticoid therapy. An interesting finding in our patient was that in addition to anti-insulin antibodies, normal C peptide, and fasting insulin, clinical signs of insulin resistance and high insulin requirements, hypotriglyceridemia was observed, which is suggested as another variable to consider for the diagnosis [1].

The treatment includes two main goals: glycemic control, and immunosuppression when it is required. No single protocol is established, and no clinical trial has been performed in this population. The treatment has certain details which must be considered, such as mean insulin requirement which was up to 5600 U/day in the NIH cohort; some patients required up to 30,000 U/day. Concentrated insulin products may improve insulin administration when higher doses are required. U-500 insulin is five times more concentrated than U-100 insulin [34]; therefore, it is considered the cornerstone of therapy in patients with insulin resistance; unfortunately, it is not available in Colombia. In a nine-study meta-analysis, patients with different types of U-100 and U-500 insulin were included: patients treated with U-500 insulin had a higher weight gain of 4.4 kg, but no difference was observed in hypoglycemias [35]. To administer such high insulin doses safely, the patient must be admitted and an intravenous insulin drip must be started. Treatment schemes have been established by academic medical centers and the main therapeutic goal is normal fasting glucose to achieve metabolic control [35, 36].

Immunosuppression is directed to resolve the underlying autoimmune process. Multiple treatment schemes have been used as was observed in the systematic review by Martins et al. [2]. NIH proposed a standardized treatment regime with a combination of rituximab, monthly pulse steroid therapy (dexamethasone 40 mg/day for four days), and cyclophosphamide [17]. This scheme was used in a 45-year-old patient with 20 kg unexplained weight loss, disseminated acanthosis nigricans, blood glucose higher than 500 mg/dL, anti-insulin receptor antibodies, and lack of metabolic control despite 600 daily insulin units and no improvement with prednisolone, azathioprine, or plasmapheresis. The patient finally improved after rituximab 750 mg/m^2^ divided into two doses two weeks apart, associated with oral cyclophosphamide 100 mg/day and dexamethasone 40 mg/day for 4 days. Fasting glucose improved down to 80–100 mg/dL, HbA1c was reduced from 11.8% to 6.5%, and perioral, periocular, and periauricular acanthosis nigricans improved as well [37].

## 5. Conclusion

In conclusion, TBIRS in nonobese patients with de novo diabetes must be considered secondary to an autoimmune disease such as SLE, with a good metabolic response to immunosuppressant management.

## Figures and Tables

**Table 1:** Laboratory results during patient's hospital stay.

Laboratory tests	Results	Reference values
Hemoglobin	10.1	12–15.5 gr/dL
Leucocytes/neutrophils/lymphocytes	2600/1000/500	4000–11,000 × mm^3^/2000–7000/1000–3000
Platelets	236,000	200,000–500,000/mm^3^
CRP	3.01	0.01–0.82 mg/dL
ESR	14	1–20 mm/h
Aspartate aminotransferase (AST)/alanine aminotransferase (ALT)	20/18	10–40 units/L/7–56 units/L
Total bilirubin/direct bilirubin	0.66/0.22	0.1–1.2 mg/dL/<0.3 mg/dL
Alkaline phosphatase/gamma glutamyl transferase (GGT)	45/29	20–140 U/L/9–48 U/L
Albumin	4	3.4–5.4 g/dL
LDH	230	140–280 U/L
Ferritin	307	20–200 ng/mL
Na^+^/K^+^/Cl^−^/Ca^2+^/Mg	138/3.8/8.8/2.2	mEq/L
Triglycerides	50	<150 mg/dL
PT/INR/PPT	10/0.9/24	10–12 s/0.9–1.15/25–35 s

*Autoimmunity laboratory*		
Antinuclear antibodies (ANA)	1 : 2560 speckled	Positive> 1 : 80
C_3_/C_4_	C_3_ 50 mg/dl C_4_ 8 mg/dl	C3 80–160 mg/dL/C4 15 a 52 mg/dL
Anti-DNA	Positive	Positive >1 : 10
Extractable nuclear antigen antibodies (ENA)	Anti Ro (+)	Positive >20 units
Antineutrophil cytoplasmatic antibodies (ANCA)	Negative	Negative
Antiphospholipid antibodies	Negative	Negative
Basal insulin	25.3	18–48 pmol/L
C peptide	30	0–4.0 ng/mL
Anti-insulin antibodies	Positive	Negative

**Table 2:** SLE-associated TBIRS patients' description.

Gender/age, race	Time- to diabetes diagnosis	Time-to resistance	ANA titer/pattern	Acanthosis nigricans	SLE characteristics	ACRI-AAI	Maximum daily insulin dose	Treatment	Final outcome
1. *F*, 62 years, African American. Present case	3 months	3 months (catabolic symptoms)	1 : 2560 speckled	Yes	Raynaud's phenomenon Lymphopenia Hypocomplementemia	AAI	2400 U daily dose	Pulse steroid + prednisolone	Remission (metabolic control)
2. *F*, 47 years, Asian. [4]	3 months	3 months (catabolic symptoms)	Positive speckled	Yes	Fever Oral ulcers Weight loss Axillar lymphadenopathies Leukopenia	ACRI	12,000 U daily dose	Pulse steroid + 20 mg for 4 weeks	2 years remission
3. *F*, 60 years, African American. [8]	1 year (hypoglycemia)	3 months (catabolic symptoms)	Not reported	Yes	Not reported	ACRI	Hypoglycemias with no antidiabetic drugs (despite glucocorticoids, glucagon and octreotide)	3 immunoglobulin cycles + prednisolone 60 mg daily + azathioprine 100 mg daily	Persistant hypoglycemias (12 months)
4. *F*, 8 years, Latina. [9]	Unknown	3 months (hypoglycemia)	1 : 640	Yes	Nephritis Dermatomyositis Raynaud's phenomenon	ACRI	Hypoglycemias with doses of 1.1 U/kg/day	Mycophenolate + prednisolone + immunoglobulin + rituximab + plasmapheresis + Bortezomib	Persistant hypoglycemias (12 months)
5. *F*, 39 years, African American. [10]	6 months (catabolic symptoms)	1 month (hypoglycemia)	1 : 2560 speckled	Yes	Antiphospholipid antibodies	AAI	1500 U daily	Rituximab + pulse high dose dexamethasone each 4 months for 4 days + maintenance with azathioprine	Remission (12 months follow-up)
6. *M*, 63 years, Asian. [11]	4 months (catabolic symptoms)	4 months (uncontrolled hyperglycemia)	1 : 320	Yes	Nephritis Cryoglobulinemia	ACRI	306 U daily	Methylprednisolone 40 mg/day + cyclophosphamide	Remission (unknown follow-up)
7. *M*, 60 years, Asian. [12]	Unknown	Unknown	1 : 2560 speckled	No	Ataxia photosensitivity Thrombocytopenia Lymphopenia Hypocomplementemia	ACRI	Continuous infusion 9 mU/kg/min	Rituximab + prednisolone + cyclosporine	Remission (unknown follow-up)—nephrotic syndrome development
8. *M*, 44 years, Asian. [13]	1 year (hyperglycemia)	Unknown (hypoglycemia)	Positive—unknown titers	Yes	Polyarthralgias Hypocomplementemia Hypergammaglobulinemia	ACRI	Hypoglycemias with no antidiabetic drugs	Rituximab	Remission (unknown follow-up)
9. *F*, 46 years, Asian. [14]	No diagnosis	3 months	Positive—unknown titers	No	Raynaud's phenomenon Thrombocytopenia Hypocomplementemia Nephritis	Negative	600 U daily	Immunoglobulin + cyclophosphamide + leflunomide	Remission (unknown follow-up)
10. *F*, 38 years, Asian[15]	Unknown	Unknown (hypoglycemias)	Positive—unknown titers	No	Photosensitivity Interstitial lung disease	ACRI	Hypoglycemias with no antidiabetic drugs	Prednisolone 30 mg/day + chloroquine 300 mg/day + azathioprine 50 mg/day	Remission (unknown follow-up)
11. *F*, 38 years, Caucasian. [16]	Unknown	2 months (renal failure and catabolic symptoms)	Positive 1 : 1280 speckled	Yes	Nephritis Malar rash Myalgias Arthralgias	ACRI	2400 U daily	Mycophenolate + pulse steroids + plasmapheresis + rituximab + IV immunoglobulin	Persistant hypoglycemia with glucose supplement requirement and continuous enteral nutrition
12. *F*, 50 years, African American. [17]	Unknown	Unknown	Positive	No	Unknown	ACRI	1300 U daily	1 rituximab cycle + 3 pulse steroid	Remission (16 months follow-up)
13. *M*, 62 years, African American. [17]	Unknown	Unknown	Positive	No	Antiphospholipid antibodies	ACRI	1250 U daily	2 rituximab cycles + 3 pulse steroid	Remission (12 months follow-up)
14. *M*, 64 years, Caucasian. [17]	Unknown	Unknown	Positive—unknown titers	No	Unknown	ACRI	1800 U daily	2 rituximab cycles + 5 pulse steroid	Remission (3 months follow-up)
15. *M*, 59 years, Asian. [18]	No diagnosis	1 month (catabolic symptoms)	Positive 1 : 80 speckled	No	Raynaud's phenomenon arthritis Alveolar hemorrhage Lymphadenopathies Pancytopenia with AIHA Hypocomplementemia	ACRI	1800 U daily	3 Cyclophosphamide cycles + prednisolone + Cyclosporine + Metformin	Remission (15 months follow-up)
16. *M*, 37 years, Asian. [19]	Unknown	3 months (hypoglycemias)	Positive—unknown titers	No	Oral ulcers Fever Arthralgias Photosensitivity Skin lesions	ACRI AAI	Hypoglycemias with no antidiabetic drugs	Prednisolone 60 mg/day + chloroquine 250 mg/day + cyclophosphamide 600 mg every 2 weeks for 6 months	Remission (unknown follow-up)
17. *F*, 50 years, from India. [20]	No diagnosis	Unknown (catabolic symptoms)	Positive 1 : 100 homogeneous	Yes	Oral ulcers Weight loss Fatigue Anemia arthritis Skin lesions	ACRI	2000 U daily	Methylprednisolone pulse + prednisolone + Azathioprine	Remission (unknown follow-up)
18. *F*, 23 years, Asian. [21]	No diagnosis	Unknown (catabolic symptoms)	Positive 1 : 320 speckled	Yes	Nephritis Hypocomplementemia	ACRI	Unknown	Methylprednisolone pulse + prednisolone	Initial remission Death due to P. jirovecii pneumonia
19. *F*, 13 years, African American. [22]	No diagnosis	3 months	Unknown	No	Unknown	ACRI	450 U daily rosiglitazone metformin	Prednisolone 60 mg daily	Unknown
20. *F*, 40 years, African American. [23]	No diagnosis	1 month (asymptomatic hyperglycemia)	Unknown	Yes	Nephritis	ACRI	4500 U daily	Methylprednisolone pulse + cyclophosphamide	Remission (unknown follow-up)
21. *F*, 37 years, African American. [24]	No diagnosis	6 months	Positive—unknown titers	Yes	Arthritis Raynaud's phenomenon	ACRI	3000 U daily	Methylprednisolone pulse + Prednisolone + 5 plasmapheresis cycles + 6 cyclophosphamide cycles	Remission (11 months follow-up)
22. *F*, 27 years, African American. [25]	No diagnosis	Unknown	1 : 640	Yes	Pericarditis Arthritis	ACRI	1200 U daily	2 cyclophosphamide cycles + prednisolone + maintenance with mycophenolate mofetil	Remission (unknown follow-up)
23. *F*, 50 years, Asian. [26]	Unknown	1 month	1 : 2560 homogeneous	Yes	Hypocomplementemia	ACRI	610 U daily	Prednisolone 30 mg + IFG-1	Remission (unknown follow-up)
24. *F*, 16 years, Latin. [27]	Unknown	2 months (catabolic symptoms)	1 : 1280	Yes	Nephritis Pancytopenia Serositis	ACRI	Unknown	Methylprednisolone pulse + 6 cyclophosphamide cycles	Remission (24 months follow-up)
25. *M*, 69 years, Asian. [28]	No diagnosis	13 months (hypo and hyperglycemia)	1 : 1280 speckled	Yes	Raynaud's phenomenon Hypocomplemententemia Nephritis	ACRI	Unknown	2 methylprednisolone pulses + Cyclophosphamide + prednisolone 30 mg daily	Remission (unknown follow-up)
26. *F*, 59 years, Caucasian. [29]	No diagnosis	15 months (catabolic symptoms and hypoglycemias)	Positive speckled (no titers)	No	Hypocomplementemia Leukopenia	ACRI	Unknown	Prednisolone for 3 months (80–40 mg/day)	Remission (unknown follow-up)
27. *F*, 24 years, Caucasian. [30]	No diagnosis	9 months (hypoglycemias)	Positive 1 : 64	No	Discoid lupus Lymphadenopathies Fever Serositis Arthritis leukopenia Lymphopenia Hypocomplementemia	ACRI	Unknown	Prednisolone for 3 months (20–5 mg/day)	Remission (unknown follow-up)
28. *F*, 52 years, African American. [31]	No diagnosis	1 month (hypoglycemias)	Positive 1 : 2084 homogeneous	No	Arthritis Alopecia Hypocomplementemia Leukopenia	ACRI	Hypoglycemias with no antidiabetic drugs despite of daily 300 gr glucose intravenous infusion	Prednisolone (120–10 mg/day)	Remission (unknown follow-up)
29. *F*, 49 years, African American. [32]	2 years	14 months (catabolic symptoms)	Positive 1 : 160 speckled	Yes	Alopecia Leukopenia Nephritis	ACRI	24,000 daily	prednisone	Death (unknown cause)
30. *F*, 40 years, African American. [32]	No diagnosis	Unknown (catabolic symptoms)	Positive 1 : 360 speckled	Yes	Arthralgias Leukopenia Nephritis Neurolupus	ACRI	6000 UI daily	Pulse steroids	Death (unknown cause)
31. *F*, 23 years, African American. [32]	Unknown	9 months (catabolic symptoms)	Positive—unknown titers	Yes	Arthritis Leukopenia Hypocomplementemia Nephritis	ACRI	2600 U daily	3 plasmapheresis and lymphapheresis cycles + 3 cyclophosphamide cycles + Prednisone	Remission (unknown follow-up)
32. *F*, 51 years, African American. [33]	4 years	8 months (catabolic symptoms)	Positive 1 : 160 speckled	Yes	Alopecia Hypocomplementemia	No tested	25,000 U daily	No drugs	Spontaneous remission (20 months follow-up)

**Table 3:** SLE-associated TBIRS patients' characteristics.

Clinical and demographic characteristics	Mean and frequencies
Race	African American 39% Caucasian 16% Asian 39% Latin 6%

Age	Mean: 43 years Range: 8–69 years

Hypoglycemia	35%

Acanthosis	61%

Hypocomplementemia	39%

Late onset lupus (≥50 years)	37.5%

ANAs	Speckled: 32% Homogeneous: 10% No specification/no report: 58%

Treatment	Steroids: 84% Rituximab: 26% Azathioprine: 13% Mycophenolate: 13% Immunoglobulin: 10% Cyclophosphamide: 10% Methotrexate: 10% cyclosporine: 6% Bortezomib: 3%

Treatment response	Spontaneous 3.1% Unknown 17.9% Remission 80%

Death	10% (4 patients) 2 due to unknown causes 1 due to P. jirovecii infection 1 due to motor vehicle accident
